# Hypoglossal canal: an osteological and morphometric study on a collection of dried skulls in an Italian population: clinical implications

**DOI:** 10.1186/s40001-023-01489-6

**Published:** 2023-11-08

**Authors:** Massimo Guarna, Paola Lorenzoni, Daniela Franci, Margherita Aglianò

**Affiliations:** Department of Medical, Surgical and Neurological Sciences, University of Siena, Via Aldo Moro 6, 53100 Siena, Italy

**Keywords:** Hypoglossal canal, Hypoglossal canal variations, Morphometry, Occipital condyle, Skull base

## Abstract

**Background:**

The hypoglossal canal is a dual bone canal at the cranial base near the occipital condyles. The filaments of the hypoglossal nerve pass through the canal. It also transmits the meningeal branch of the ascending pharyngeal artery, the venous plexus and meningeal branches of the hypoglossal nerve. The hypoglossal nerve innervates all the intrinsic and extrinsic muscles of the tongue except the palatoglossal and is fundamental in physiological functions as phonation and deglutition. A surgical approach to the canal requires knowledge of the main morphometric data by neurosurgeons.

**Methods:**

The present study was carried out on 50 adult dried skulls: 31 males: age range 18–85 years; 19 females: age range 26–79 years. The skulls came from the ''Leonetto Comparini'' Anatomical Museum. The skulls belonged to people from Siena (Italy) and its surroundings (1882–1932) and, therefore, of European ethnicity. The present study reports (a) the osteological variations in hypoglossal canal (b) the morphometry of hypoglossal canal and its relationship with occipital condyles. One skull had both the right and left hypoglossal canals occluded and, therefore, could not be evaluated. None of the skulls had undergone surgery.

**Results:**

We found a double canal in 16% of cases, unilaterally and bilaterally in 2% of cases. The mean length of the right and left hypoglossal canals was 8.46 mm. The mean diameter of the intracranial orifice and extracranial orifice of the right and left hypoglossal canals was 6.12 ± 1426 mm, and 6.39 ± 1495 mm. The mean distance from the intracranial end of the hypoglossal canal to the anterior and posterior ends of occipital condyles was 10,76 mm and 10,81 mm. The mean distance from the intracranial end of the hypoglossal canal to the inferior end of the occipital condyles was 7,65 mm.

**Conclusions:**

The study on the hypoglossal canal adds new osteological and morphometric data to the previous literature, mostly based on studies conducted on different ethnic groups.The data presented is compatible with neuroradiological studies and it can be useful for radiologists and neurosurgeons in planning procedures such as transcondilar surgery. The last purpose of the study is to build an Italian anatomical data base of the dimensions of the hypoglossal canal in dried skulls..

## Introduction

The hypoglossal canal (HC) is a dual bone channel that is located at the cranial base near the occipital condyles (OC) and it is called the anterior condylar canal. The HC is directed anterolateral and localized above the OC at its junction of anterior 1/3 and posterior 2/3. The filaments of the hypoglossal nerve pass through the canal, accompanied by a wide venous plexus, an emissary vein [EV] to the transverse sinus and a branch of the ascending pharyngeal artery [APA]

[11, 26]. The hypoglossal nerve innervates all the intrinsic and extrinsic muscles of the tongue except the palatoglossal and is fundamental in physiological functions such as phonation and deglutition. Morphometric studies have been particularly useful for antropologists who, by measuring the width of the canal, have assumed a greater development of the size of the hypoglossal nerve in hominids than apes and the most primitive hominids [7, 14].

Thus, human vocal abilities may have appeared much earlier in time than the first archaeological evidence for symbolic behavior[14]. Nonetheless, the hypothesized link between hypoglossal canal size and speech remains unproved [7].

Various pathologies of the skull base such as fractures, gunshot injury [19, 20] neoplasms affect the hypoglossal canal and nerve and a surgical approach to the canal requires knowledge of the main morphometric data by neurosurgeons. In the transcondylar surgical approach, which allows a more direct approach to the pontomedullary structures, a more or less extensive trepanation of the OC is performed [1, 9]. Therefore, it is necessary to know the morphometric relationships between the OC and the HC which is in anatomical relationship with it.[8, 9, 12, 15, 16, 22, 25]

The morphology of the canal is variable, being present inside the canal, spicules or even bone bridges that can lead to nerve entrapment during ossification of the occipital bone, causing alterations in speech. The present study reports the percentages of variations in HC: we observed the skulls carefully.

- To note variations in the HC or the percentages of double HC, whether it was unilateral or bilateral, and if there were differences related to sex;
- To study the morphology of HC and its relationship with the surrounding bone structures, in particular OC. These data are particularly important in the neurosurgical transcondylar approach to the posterior cranial fossa [9]. The last purpose of the study is to build a normal Italian anatomical data base of the dimensions of the HC of dried skulls.

## Materials and methods

We carried out the study on 50 undamaged dry adult human skulls of both sexes and known age (31 males: age range 18–85 years; 19 females: age range 26–79 years), collected from local museum Leonetto Comparini of Siena. The skulls belonged to people from Siena and its surroundings (1882–1932). The approval of the ethics commission was not requested because the skulls have been the property of the Anatomical Museum for many years.

We observed the variations in the morphology of the canal and, in particular, the presence of a bony spur or a bony bridge or septum. The different forms of subdivisions of HC are classified into four types. There are various classifications in osteology and anatomical variations of the hypoglossal canal [2, 16, 24]. We have classified the different forms of subdivisions of HC into four types.

Type 1: no bony spine or septum.

Type 2: bony spine on the outside (2A) or the inside (2B) border or in the centrum (2C) of the canal.

Type 3: incomplete septum that divides a portion of the canal into two parts.

septum present near the external opening of the canal (3A).

septum present near the internal opening of the canal (3B).

septum in the central part of the canal (3C).

Type 4: presence of a central complete septum that divides the canal into two parts.

septum present near the external opening of the canal (4A).

septum present near the internal opening of the canal (4B).

septum in the central part of the canal (4C).

We employed a digital Vernier caliper to measure the length of the canal, the diameter of the internal and external orifices of the canal and the distance of HC from OC. All measurements are expressed in mm. The digital caliper had the following technical characteristics: measuring range: 0–150 mm; resolution: 0.01 mm; accuracy: ± 0.02 mm; measurement repeatability: ± 0.01 mm. Each measurement was repeated twice and an average was obtained. More precisely, the measured parameters were the following:

External diameter.

Internal diameter.

Length.

Distance between the anterior border of the internal hole of HC and the anterior border of CP on the same side (Dist. I-A).

Distance between the posterior edge of the internal hole of HC and the posterior edge of the CP on the same side (Dist. I-P).

Distance between the lower edge of the internal hole of HC and the inferior edge of the CP on the same side (Dist. I-I).

To measure the length of the canal, we used a rigid plastic wire that we inserted inside the channel to match one end with the edge of an orifice, then with very thin pliers we clamped the end of the wire in correspondence with the margin of the other orifice. Afterwards, we removed the wire from the hole, stretched it and measured the section of interest using the digital caliper.

Using the means, descriptive statistical data (minimum, maximum, median value) were calculated for each parameter.

## Statistical analysis

The data of each parameter of the right HC were compared with those of the left HC using a Student's t-test. The normality of the data distribution was assessed with the Shapiro–Wilk test.

In addition, the data of each parameter of the right and left HC of the skulls belonging to males and those belonging to females were compared using a Student’s t-test.

Statistical significance was accepted at *P* < 0.05.

We employed the software GraphPad Prism 6 for statistical analysis.

## Results

Percentages of channel types:

66% were type 1.

19% type 2B.

1% type 2C.

2% type 3B.

10% type 4B.

2% were occluded and therefore not assessable.

**Fig. 1:**
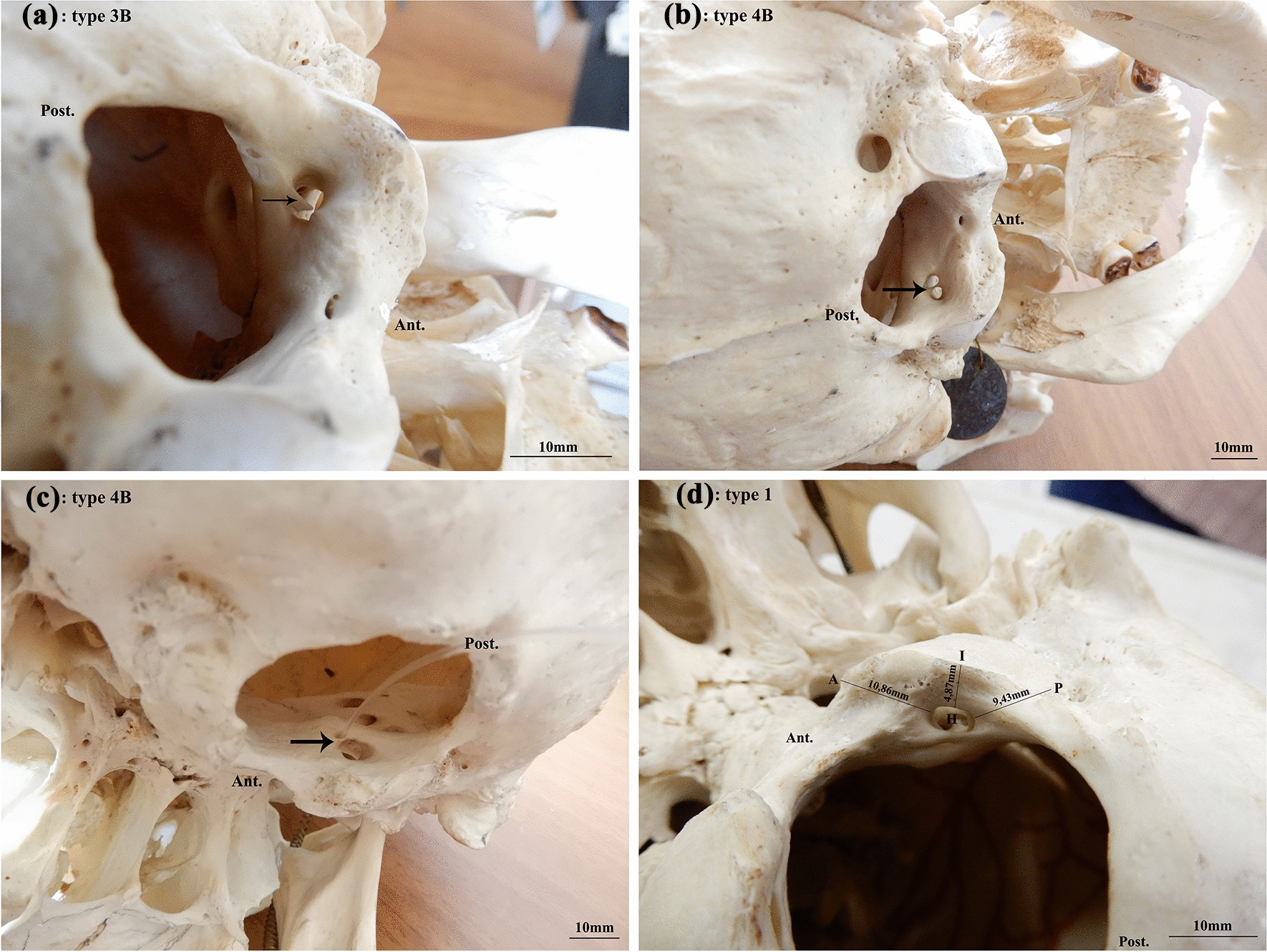
**a** hypoglossal canal with incomplete septum. The figure shows the right hypoglossal canal of a 40-year-old female. The canal is of type 3B, i.e., it has an incomplete septum. The arrow indicates the incomplete bony septum inside the canal. **b** hypoglossal canal with complete septum. The figure shows the left hypoglossal canal of a 40-year-old female. The canal is of type 4B, that is, it has a complete septum. The arrow indicates the bony septum that divides the canal into two symmetrical parts. **c** hypoglossal canal with complete septum. The figure shows the right hypoglossal canal of a 48-year-old female. The canal is of type 4B that is, it has a complete septum that divides it into two halves of very different caliber. This skull on the left presented a 4B canal with 2 equivalent holes. **d** distances of the internal hole of the hypoglossal canal from the anterior, posterior and inferior edges of the condylar process. The figure shows the distances between the inner edge of the hypoglossal canal and the anterior (IA), posterior (IP) and inferior (II) edges of the condyloid process

Right HC: 34 were Type 1, 10 were Type 2 B, 1 was Type 2C, 1 was Type 3B, (Fig. 1a) 3 were Type 4B, 1 was occluded, therefore, not evaluable (Table 1).

**Table 1:** Types of right hc

Types right hyp	Number	%
Type 1	34	68
Type 2B	10	20
Type 2C	1	2
Type 3B	1	2
Type 4B	3	6
Not evaluable	1	2

The tables show the number and the percentages for each type of channel for side. The values on the right were compared with those on the left with the Student's t-test, they were not statistically different, p-value > 0.999

Left HC: 32 were Type 1, 9 were type 2B, 1 was Type 3B,7 were Type 4B,1 an occluded one was not evaluable (Table 2).

**Table 2:** Types of left hc

Types left hyp	Number	%
Type 1	32	64
Type 2B	9	18
Type 3B	1	2
Type 4B	7	14
Not evaluable	1	2

The tables show the number and the percentages for each type of channel for side. The values on the right were compared with those on the left with the Student's t-test, they were not statistically different, *p*-value > 0.999

Bipartite HCs were found on the left side alone in 6 skulls, on the right side alone in 2 and on.

both sides in 1. A bipartite hypoglossal canal was, therefore, found in 16% of cases unilaterally and in 2% bilaterally Fig. 1b, c).

The subsequent morphometric data are shown in Table 3.

**Table 3:** With the averages of the measured parameters of the right and left hc and the significance of the differences

Parameters	MEAN (mm)	SD	Med	Min Max	*P*-values	Statistical significance
Length of right HC	8,67	1,86	8,66	4,65 14,69	0,25	Not significant
Length of left HC	8,26	1,67	8,24	5,51 14,58		
Ex end diameter right Hc	6,33	1,40	6,16	4,06 12,67	0,71	Not significant
Ex end diameter left Hc	6,44	1,59	6,20	4,16 12,96		
Int end diameter right HC	6.03	1,42	5,69	3,34 13,06	0,53	Not significant
Int end diameter left Hc	6,21	1,43	5,90	4,15 11,04		
Distance I-A right	10,65	1,48	10,87	6,5 13,62	0,47	Not significant
Distance I-A left	10.88	1.70	11,10	7,07 14,20		
Distance I-P right	11,11	1,58	11,08	8,19 15,23	0,052	Almost significant
Distance I-P Left	10,51	1,47	10,35	7,67 13,57		
Distance I-I right	7,85	1,73	7,77	3,25 11,64	0,22	Not significant
Distance I-I LEFT	7,45	1,52	7,38	3,85 11,54		

The data of each parameter of the right HC were compared with those of the left HC using a Student's t-test

The I-P distance is greater to the right than to the left and the difference comes very close to significance

Distance between the internal hole of HC and the anterior edge of the CP on the same side (Dist.

I-A) was 10,76 mm on both sides.

Distance between the internal hole of HC and the posterior edge of the CP on the same side (Dist.

I-P) was 10,81 mm on both sides.

Distance between the internal hole of HC and the inferior edge of the CP on the same side (Dist. I-I) was 7,65 mm on both sides (Fig. 1d).

The mean length of HC was measured as 8,67 ± 1,86 mm on the right side and 8,26 ± 1,67 mm on the left side.

The external diameter was 6,33 ± 1,40 on the right side and 6,44 ± 1,59 mm on the left side.

The internal diameter was 6,03 ± 1,42 on the right and 6,21 ± 1,43 on the left side.

Then the data of each parameter relating to the right HC of the male group were compared with those relating to the right HC of the female group: no value was statistically significant.

Finally, the data of each parameter relating to the left HC of the male group were compared with those relating to the left HC of the female group: the distance I-I of the males (mean 7,87 ± 1,60) compared to that of the females (mean 6,76 ± 1,13) was statistically significant (*P* = 0.011). Furthermore, the IP distance (mean male 10,80 ± 1,60; mean female 10,03 ± 1,09) revealed a trend towards significance (*P* = 0.075). The comparative morphometric data between males and females regarding the distance between the internal hole of the HC and the ends of the OC are shown in Table 4.

**Table 4:** Distance between the front edge of the inner hole of the hc and the front, rear and lower margin of the condyloid process in the both sides and in the two sexes

Parameters	Mean (mm)	SD	Med	Min Max	*P*-values	Statistical significance
Distance I-A right male	10.74	1.18	10,88	6,5 12,62	0,56	Not significant
Distance I-A right female	10,49	1,89	10,55	7,01 13,62		
Distance I-A left male	11.18	1,47	11,18	8,15 14,20	0,10	Not significant
Distance I-A left female	10,38	1,96	11,08	7,07 13,30		
Distance I-P right male	11,31	1,49	11,27	8,54 15,16	0,25	Not significant
Distance I-P right female	10,78	1,69	10,58	8,19 15,23		
Distance I-P left male	10,80	1,60	10,41	7,85 13,57	0,07	Not significant
Distance I-P left female	10,03	1,09	10,22	7,67 11,53		
Distance I-I right male	8,02	1,76	8,19	3,25 10,84	0,39	Not significant
Distance I-I right female	7,58	1,67	7,48	5,11 11,64		
Distance I-I left male	7,87	1,60	7,76	3,85 11,54	*P* = 0,011	Significant
Distance I-I left female	6,76	1,13	7,18	4,97 8,55		

The data of each parameter of the right and left HC of the skulls belonging to males and those belonging to females were compared using a Student’s t-test

The distance I-I to left is larger in the male than in the female and this difference is significant

(*p* = 0, 011)

## Discussion

Detailed knowledge of the osteology and morphometry of the HC is necessary for the neurosurgeon to plan any surgical interventions in the area and cut morbidity and mortality rates [12, 13]. The anatomical variations of the HC are important also for anthropologists who can infer possible ethnicity differences from various studies [4] and for anatomysts, radiologists [11] and for forensic sciences. The variations in the HC division have been the subject of studies conducted on dried skulls or with neuroradiological methods [11]. In our study, we found a double unilateral HC channel in 16% of cases (7 on the left and 3 on the right) and bilateral in 2% of cases. Nearly similar results were reported in a neuroradiological study with multislice TAC in a Japanese population where double hypoglossal canal was identified in 16.9% of the patients, and was bilateral in 2.2% [11]. Osteological studies on skulls belonging to various ethnicities show some differences, regarding the duplication of the hypoglossal canal by a more or less complete septum. Kumar et al. [15] in an Indian population of 50 dried skulls did not observe complete septa. Meera et al. [17] in 60 skull bones observed double HC in 12 skulls (20%) out of which 4 were bilateral and 8 were unilateral. Bhuller et al. [5] found the HC divided into two canals by small bony spicules in 28.12% of cases. Yadav et al. [28] observed bilateral duplicated HC in 3 skulls (3.75%) while unilateral duplication was seen in 15 skulls (18.75%). Finally complete osseous bridging, in the outer or inner part of the canal, was visible in 19.83% of specimens (46/23) as reported by Paraskevas et al. [22]. In a large sample of dried skulls (585 skulls) Berry and Berry [4] discovered a double hypoglossal canal in 14.6%

An endocranial approach was used by Ogut et al. [21] to study the morphology of the hypoglossal canal on 18 formalin-fixed cadaver heads, recognizing five types of canal depending on whether they were unique, divided by a meningeal septum or a bony septum. The most observed type was Type 3:52,94,4% (left) and 25% (right) In this type,two different nerve output from endocranial HC and rootlets from hypoglossal nerve were separated by an osseus septum. The anatomical division of the canal creates compartments that can compress the EV,the APA or the hypoglossal nerve [15,]

Double hypoglossal canal is important clinically, it may trap the hypoglossal nerve during ossification of occipital bone The entrapment of CN XII roots due to the incomplete ossification of the occipital bone or variational.

types cause difficulties in speech, atrophy of the tongue, and compression of the venous plexus [15, 24], and and its recognition is important to neurosurgeons,neuroradiologists and anthropologists [0.9,11,14].

In the present study, the mean length of the canal was 8,67 ± 1,86 mm on the right side and 8,26 ± 1,67 mm on the left side and this difference was similar to the value reported by Lyrtzis et al. [16], on 141 Greek adult dried skulls (8,89 mm on right side -9,03 mm on left side) and by Berlis et al. [3], who in a correlative study between CT scan and 60 dried skulls, found a length of 7.78 mm. The diameter of the extracranial end of the right HC in our study was 6,33 mm and the left one was 6,44. The difference between the two sides was not significant. The diameter of the intracranial end of the right HC was 6,03 mm and 6,21 mm was the diameter of the left one;again,the difference in diameter between the two sides was not significant. Our results came close to those of Muthukumar et al. [18], who on 50 dried skulls found a diameter of the extracranial end of 7,9 mm and an intracranial end of 7,2 mm. According to Kumar et al. [15], the mean diameter of HC at its intracranial and the extracranial end was 7.48 and 7.59 mm. Very similar to our results were those obtained by Farid et al. [8] in70 Egyptian skulls: the mean diameter of the intracranial end of the right HC was 6.24 mm and the left one was 6.04 mm. However, the mean diameters of the extracranial ends were 6.18 mm and 6.04 mm. Therefore, for this important parameter, which is the length of the canal, no great differences seem to emerge between the skulls belonging to different ethnicities. The hypoglossal canal can be involved in various pathologies that require the intervention of a neurosurgeon. In these cases, different techniques are used which require the morphometry of the hypoglossal canal. A posterolateral approach to the foramen magnum and transcondylar, supracondylar, and paracondylar approaches are employed for the lower clivus, craniovertebtral junction, hypoglossal canal, and mastoid [9]. Transcondylar surgery has gained great popularity in the last time as an access route to ventral lesions in the brainstem and cervicomedullary region [25]. During transcondylar resection,the posterior one-third of OC is resected. Direct visualization of the spinal cord, the previous brain stem and the surface of the tumor can be achieved by OC resection, [23]. According to Spektor et al. [25] resection of the OC above the HC can improve the visual angle from 21 to 28% for the petroclival area, as well as provide an exposure increase from 28 to71% by resecting the jugular tubercle. Therefore, the distance between between OC and HC is important [9]. So the bony anatomy of Hc and its anatomical relationship with the condyles is necessary to plan a surgery [1]. The distance between the HC and the posterior border of the OC is critical. This measurement gives an indication of the largest amount of resectable condyle without entering the HC. Therefore,our study also concerned the anatomical morphometric relationships between HC and occipital condyle. For this purpose,we measured the distances between HC and the extremity of the condyle and the inferior margin of the same in both sides. Distance between the posterior edge of the internal hole of HC and the posterior edge of the CP on the same side (Dist. I-P) was 11,11 mm on the right and 10,51 on the left (*P* = 0,052) almost significant. Similar results were obtained by Kumar et al. [15] in 50 Indian dried skulls (10,66 on the right and 11,89 on the left), and by Vinay et al. [27] 12,98 mm still in Indian dried skulls. Our data are also similar to those of Pereira et al. [23], who finds a distance from the internal orifice of the hypoglossal canal to the posterior margin of OC on the right of 0.10.3 mm and on the left of 11.3 mm. Muthukumar et al. [18] and Barut et al. [1] suggested the distance between the HC-OC posterior border is 12 mm and 12.55 mm, as a safe zone of OCs drilling, during transcondylar neurosurgery, while Lyrzits [16] in a Greek study concluded in a safe zone of 8,17 mm. Wen et al. [29] reported that the distance between the posterior edge of the OC and the HC is approximately 8.4 mm and that a resection of the OC of that same amount would be sufficient for surgical exposure. Currently, medical practice has undergone tremendous advances,including radiology [10]. The preoperative study in hypoglossal canal surgery conducted on patients with three-dimensional computer tomography (3-D-CT) allows detailed anatomical information to be obtained. In the neuroradiological study conducted by Bulsara [6] on 20 patients, the distance from the upper part of the condyle was measured to the internal opening of the hypoglossal canal. was 11.0 + 1.4 mm (range 8.7–12.7 mm). In this way the amount of bone that, can be safely removed without violating the hypoglossal canal, can be determined preoperatively for each patient [6] With regard to gender differences, none were found except for the distance between the inferior edge of the internal hole and the inferior edge of the condyloid process on the same side (Dist. I-I) which was found to be greater in men than in women, 7,87 ± 1,60 mm mm and 6,76 ± 1,13 mm mm, respectively (*P* = 0,011). It is important to emphasize this gender difference that has never been described before by other authors and that may have particular importance in transcondylar surgery in the planning of surgery to cut morbility and mortality. Gender dimorphism was found in the study of Lyrtzis et al. [16], about the right length of the HC and the left distance of the HC from the posterior edge of the occipital condyles.

## Limitations

A limitation of our study is that it was conducted on dried skulls and lacks radiological findings which can be useful in planning neurosurgery in the posterior cranial fossa. Also, a greater number of skulls would probably highlight even other statistical differences.

## Conclusions

The study on the hypoglossal canal adds new osteological and morphometric data to the previous literature mostly based on studies conducted on different ethnic groups. Some differences in the measured parameters emerge from the study for each side and sex. Our morphometric data state that no size of hc shows a side asymmetry (hcs extracranial and intracranial diameters and hcs length), except the side asymmetry of the distance between the posterior edge of the internal hole of hc and the posterior edge of the oc on the same side. That was almost significant. With regard to gender differences, none were found significant except for the distance between the inferior edge of the internal hole and the inferior edge of the condyloid process. This difference observed may have particular importance in transcondylar surgery in the planning of surgery. No similar study on an Italian homogeneous skull population was found in the literature search. The data presented can provide valuable information to neuroradiologists and neurosurgeons. The dimensions of the hypoglossal canal are helpful for radiologists and neurosurgeons while intervening with cases like schwannoma of the hypoglossal nerve in the posterior cranial fossa.

Osteological data on the variation of the external and internal orifices of the hypoglossal canal and its location may be particularly useful for procedures such as condylectomy during transcondylar surgery.

Furthermore, the study highlights differences or similarities, when there are, with studies on other populations which could be useful to anthropologists.

## Data Availability

The material used comes from the museum “Leonetto Comparini “of the University of Siena.
